# Effect of method of caries induction on aged resin-dentin bond of primary teeth

**DOI:** 10.1186/s12903-015-0049-z

**Published:** 2015-07-11

**Authors:** Tathiane Larissa Lenzi, Ana Flávia Bissoto Calvo, Tamara Kerber Tedesco, Hérica Adad Ricci, Josimeri Hebling, Daniela Prócida Raggio

**Affiliations:** Department of Pediatric Dentistry, School of Dentistry, Universidade de São Paulo, Av. Prof. Lineu Prestes, 2227 – Cidade Universitária, São Paulo, SP 05508-000 Brazil; Department of Orthodontics and Pediatric Dentistry, Araraquara School of Dentistry, Universidade Estadual Paulista (Unesp), Rua Humaitá 1680, 14801-903 Araraquara, SP Brazil

**Keywords:** Caries-affected dentin, Artificial caries, pH-cycling, Microbiology, Longevity

## Abstract

**Background:**

To investigate the influence of chemical and microbiological methods of caries induction on bond degradation of adhesive systems to primary dentin.

**Methods:**

Flat dentin surfaces from 36 primary molars were assigned to three groups (n = 12) according to method to induce caries-affected dentin: (1) control (sound dentin); (2) pH-cycling; and (3) microbiological caries induction model. Teeth were submitted to caries induction for 14 days for both methods, and the sound dentin was stored in distilled water during the same period. Specimens from each experimental group were then randomly reassigned to two subgroups (n = 6) according to the adhesive system tested: two-step etch-and-rinse adhesive (Adper Single Bond 2 - SB) or two-step self-etch system (Clearfil SE Bond - CSEB). Composite buildups were constructed and sectioned to obtain bonded sticks to be subjected to microtensile (μTBS) testing immediately or after 12 months of water aging. The μTBS means were analyzed by three-way repeated measures ANOVA and Tukey’s tests (α = 0.05).

**Results:**

The μTBS values obtained to artificially-created caries-affected dentin were lower compared with sound dentin, but were not affected by method of caries induction. Water storage for 12 months reduced bond strengths, except to CSEB bonded to sound dentin.

**Conclusion:**

Chemical and microbiological methods affect similarly the stability of resin-dentin bonds in primary teeth.

## Background

Selective caries removal has been broadly advocated to preserve tooth structure and avoid unnecessary pulp tissue exposure [1]. In this way, resin composite is bonded into a prepared cavity after removal of infected dentin, in which the cavity floor frequently consists of caries-affected dentin (CAD).

CAD forms resin-bonded interfaces that are more complex than sound dentin, being composed of multiple zones: resin-infiltrated dentin, poorly-infiltrated dentin, exposed dentin and partially demineralized dentin [2]. Consequently, these sites are more prone to undergo both hydrolytic [3] and enzymatic degradation [4] over time.

Degradation of resin-dentin bonds to CAD has been poorly investigated [3, 5–7]. Generally, bond strength tests have been performed in CAD using extracted carious teeth. Nevertheless, the lack of standardization of natural caries lesions creates technical difficulties for bonding evaluations [8]. This is mainly crucial for primary teeth, so that there is little scientific information about the bonding stability of different adhesive systems to CAD of primary teeth after long-term of water storage [9].

Thereby, *in vitro* models have been used to induce caries-like lesions under controlled conditions [10–13]. Chemical methods such as pH-cycling model, provide a superficial dentin demineralization, resulting in a substrate with similar hardness compared to natural CAD [10]. Conversely, the microbiological method promotes an excessive softening of dentin, but with a more comparable morphological pattern of collagen degradation to natural caries lesions [10, 13].

It has been recently demonstrated that immediate bond strength of adhesive systems to dentin is not affected by the method of caries induction [14]. However, since the process of collagen degradation by bacterial esterase is only simulated with a microbiological model, the stability of bonded interfaces may be influenced by the method of dentin caries induction. To date; no information is available regarding its implication on bond deterioration to artificially-created CAD of primary teeth.

Therefore, this study aimed to investigate the influence of chemical and microbiological methods of caries induction on bondi longevity of an etch-and-rinse and a two-step self-etch adhesive systems to primary dentin.

## Methods

### Tooth selection and preparation

Thirty-six sound, naturally exfoliated second primary molars were obtained after the patient’s informed consent, and the study protocol was approved by the local Research Ethics Committee of University of São Paulo. The teeth were disinfected in 0.5 % aqueous chloramine and stored in distilled water at 4 °C until use.

The occlusal enamel was removed with a water-cooled diamond saw in a cutting machine (Labcut 1010, Extec Co., Enfield, CT, USA) to obtain flat mid-coronal dentin surfaces. The surrounding enamel was also removed with a diamond bur in a high-speed handpiece (# 3195, KG Sorensen, Barueri, Brazil) with water-cooling spray.

Teeth were randomly allocated into three groups (n = 12), which underwent different procedures, as follows: (1) immersion in distilled water at 37 °C during the experimental period; that is, control (sound dentin); (2) exposure to artificial caries induction with a pH-cycling model; and (3) exposure to microbiological caries induction method.

An additional 0.5 mm thick cut was made in the teeth assigned to the control and pH-cycling model groups to compensate the further removal of carious dentin in the teeth from microbiological group [12]. The dentin surfaces were carefully examined under a stereomicroscope at 30× magnification to confirm the absence of enamel islets.

The exposed occlusal dentin surfaces were then polished with 600-grit silicon carbide abrasive paper under running water for 30 s to produce a standardized smear layer. In the microbiological method group, this procedure was carried after carious tissue removal.

Artificial caries induction groups had their cervical portions sealed with epoxy resin (Araldite Hobby, Ciba Especialidades Químicas Ltda, São Paulo, SP, Brazil) and covered with two coats of acid-resistant varnish (Colorama Maybelline Ltda, São Paulo, SP, Brazil), leaving only the occlusal dentin surface exposed.

### Artificial caries induction by pH-cycling

Specimens were individually immersed in 10 mL of demineralizing solution (2.2 mM CaCl_2_, 2.2 mM NaH_2_PO_4_, 50 mM acetic acid adjusted pH of 4.8) for 8 h and in the same volume of remineralizing solution (1.5 mM CaCl_2,_ 0.9 mM NaH_2_PO_4,_ 0.15 mM KCl adjusted pH of 7.0) for 16 h [10]. This procedure was carried out for 14 days at room temperature without agitation. The solutions were renewed at each cycle.

### Microbiological caries induction

Specimens were sterilized with ethylene oxide and then transferred aseptically to a beaker containing a cariogenic solution. The solution consisted of 3.7 g of BHI broth (Brain Heart Infusion, Becton Dickinson and Company; Sparks, MD, USA), 2.0 g of sucrose (Synth, LabSynth; São Paulo, SP, Brazil), 1.0 g of glucose (Synth, LabSynth; São Paulo, SP, Brazil) and 0.5 g of yeast extract (Becton Dickinson) for every 100 ml of distilled water. This solution was autoclaved at 121 °C for 20 min prior to the inoculation of 2 % of Streptococcus mutans strain ATCC 25175 (10^8^ cfu/ml), with pH around of 4.0. The teeth were suspended by orthodontic wires into the acidic S mutans-containing solution and incubated at 37 °C in a microaerophilic jar (BBL GasPak system, Becton-Dickinson, Franklin Lakes, NJ, USA) for 14 days [15]. Every 48 h, the specimens were transferred to another beaker containing a new cariogenic solution to provide fresh substrate to the micro-organisms. The biofilms covering the teeth were removed with gauze and the teeth were again sterilized as aforementioned.

The resulting dentin was darker in color and softer, as felt with a sharp explorer held without pressure. The softened carious dentin (infected dentin layer) was then removed with 320-grit silicon carbide abrasive paper under running water (KG Sorensen; Barueri, SP, Brazil). Caries removal stopped when a layer of caries-affected dentin was reached. That layer was harder and slightly darker than the infected dentin layer. A single experience and previously trained operator performed this procedure.

### Bonding procedures

Teeth from each experimental group were randomly reassigned to two subgroups according to the adhesive system tested (Adper Single Bond 2 - SB and Clearfil SE Bond - CSEB). Adhesives were applied strictly in accordance with the respective manufacturer’s instructions, described in Table 1.

**Table 1:** Materials, manufacturers, components, batch numbers and application mode of adhesive systems tested

*Adhesive system (manufacturer)*	*Main components*	*Batch number*	*Application mode*
SB	Etchant : 35 % phosphoric acid	N187625	1. Apply etchant for 15 s
			2. Rinse for 10 s
	HEMA, water, ethanol, Bis-GMA, dimethacrylates, amines, metacrylate-functional copolymer of polyacrylic and polyitaconic acids, 10 % by weight of 5 nanometer-diameter spherical silica particles	N190766BR	3. Blot excess water
			4. Apply 2 consecutive coats of adhesive for 15 s with gentle agitation
Adper Single Bond 2 (3 M ESPE, St. Paul, MN, USA)			
			5. Gently air dry for 5 s
			6. Light-cure for 10 s
CSEB	*Primer:* MDP, HEMA, hydrophilic dimethacrylate, dl-campherquinone ,N,N-diethanol-*p*-toluidine, water	00955A	1. Apply primer on dry dentin surface and left undisturbed for 20 s
Clearfil SE Bond			
		01416A	2. Dry with air stream for 5 s to evaporate the volatile ingredients
(Kuraray Medical Inc., Tokyo, Japan)	*Bonding:* MDP, Bis-GMA, HEMA, hydrophobic dimethacrylate, dl-campherquinone, N,N-diethanol-*p-*toluidine, silanated colloidal silica		
			3. Apply bond and gently air dry
			4. Light-cure for 10 s

*MDP* 10-methacryloyloxydecyl-dihydrogen-phosphate; *Bis-GMA* bisphenyl-glycidyl methacrylate; *HEMA* 2-hydroxyethyl methacrylate)

After the bonding procedures, resin composite (Filtek Z250, shade A3, 3 M ESPE, St. Paul, USA) was built up on the bonded surfaces in increments of approximately 1.5 mm, and individually light-cured for 20 s with a halogen light-curing unit (Jetlite 4000 Plus, J. Morita USA inc., CA, USA). Light intensity output was monitored with a Demetron Curing Radiometer (Demetron Research Corporation, Danbury, CT, USA) and was at least 600 mW/cm^2^. The bonded specimens were stored in distilled water at 37 °C for 24 h.

### Microtensile bond strength (μTBS)

Teeth were sectioned longitudinally in the mesio-distal and buccal-lingual directions across the bonded interface using a water-cooled diamond saw in a cutting machine (Labcut 1010, Extec Co., Enfield, CT, USA) to obtain sticks with a cross-sectional area of approximately 0.8 mm^2^. The cross-sectional area of each stick was measured with a digital caliper (Absolute Digimatic, Mitutoyo, Tokyo, Japan) for calculating bond strength (in MPa). The sticks were carefully examined with a stereomicroscope at 30 × magnification and those with defects at the resin-dentin interface were discarded.

The bonded sticks originating from the same teeth were randomly subdivided into 2 groups, according to storage period – immediately (24 hours) or for 12 months (in distilled water containing 0.4 % sodium azide at 37 °C). The storage solution was not changed and its pH was monitored monthly. After each storage period, the bonded sticks were attached to a device for microtensile testing with cyanoacrylate resin (Zapit, Dental Ventures of America, Corona, CA, USA) and subjected to microtensile test on a universal testing machine (Kratos Dinamômetros, São Paulo, SP, Brazil) at a crosshead speed of 1 mm/min.

### Failure mode

After the test, both sides of all debonded sticks were observed in a stereomicroscope (HMV II, Shimadzu, Kyoto, Japan) at 400× magnification to determine failure mode: adhesive/mixed (failure at the resin-dentin interface or mixed with cohesive failure of the neighboring substrate) or cohesive (failure exclusively within the dentin or resin composite). Pre-testing failures due to specimens’ preparation or water storage time were also recorded.

### Statistical analysis

The experimental unit in this study was the tooth. Half of the specimens of each tooth were tested after 24 hours of water storage and the other half was tested after 12 months. Thus, mean of the μTBS (MPa) of all sticks from the same hemi-tooth was averaged for statistical purposes. The μTBS mean for every testing group was expressed as the average of the 6 hemi-teeth used per group. The prematurely debonded specimens were included in the hemi-tooth mean with an arbitrary value of 4.0 MPa (mean value between zero and the minimum bond strength value observed in this study) [16].

Normal distribution of bond strength data and equality of variances were assumed after Kolmogorov-Smirnov and Levene’s tests, respectively. The μTBS means were subjected to three-way repeated measures analysis of variance (method of caries induction *vs.* adhesive system *vs.* storage period) and Tukey *post hoc* test for pair-wise comparisons. The clustered variable was the storage period.

The Chi-square (*X*^2^) test was used to compare failure mode among the experimental groups and pre-testing failures, considering the specimen as experimental unit for this analysis. The significance level was set at p < 0.05. Statistical analysis was performed with GMC software, version 7.7 (FORP USP, Ribeirão Preto, SP, Brazil).

## Results

The microtensile bond strength means (MPa) and standard deviations for all experimental groups are displayed in Table 2. The main factors “method of caries induction” (p <0.01; F = 119.25), “storage period” (p <0.01; F = 92.39) as well as the cross-product interactions “adhesive system *vs.* storage period” (p = 0.04; F = 4.33) and “adhesive system *vs.* storage period *vs.* method of caries” (p = 0.03; F = 6.80) were statistically significant.

**Table 2:** Microtensile bond strength (MPa) means and standard deviations for all experimental groups (*)

*Storage period*	*24 hours*		*12 months*	
*Method vs. Adhesive*	Adper Single Bond 2	Clearfil SE Bond	Adper Single Bond 2	Clearfil SE Bond
Control (sound dentin)	44.2 ± 6.8 ^A,a^	41.0 ± 6.5 ^A,a^	25.5 ± 5.7 ^A,b^	36.4 ± 4.6 ^A,a^
pH-cycling	23.3 ± 6.3 ^B,a^	25.0 ± 5.0 ^B,a^	10.3 ± 3.7 ^B,b^	9.6 ± 3.4 ^B,b^
Microbiological	18.3 ± 4.9 ^B,a^	17.8 ± 4.4 ^B,a^	7.6 ± 2.1 ^B,b^	8.9 ± 1.7 ^B,b^

(*)Different superscript capital letters indicate significant differences between columns. Different superscript lower case letters indicate statistically significant differences between rows (p <0.05)

Both methods of caries induction resulted in lower μTBS values than those obtained for sound dentin, without difference between them. Overall, 12 months of water aging resulted in a significant reduction in bond strength values (decreasing ranged of 42.3 % to 61.6 %), except when self-etch adhesive system was bonded to sound dentin (it was around 11 %).

The failure mode and the percentage of pre-testing failures observed are showed in Table 3. For all groups, adhesive/mixed failure prevailed. The percentage of cohesive in dentin fracture was higher in control groups compared with microbiological groups, irrespective of adhesive system. Pre-testing failures were more frequent in microbiological groups after 12 months aging.

**Table 3:** Number and percentage of specimens (%) in according with failure mode, pre-testing failures and total of specimens obtained for experimental groups. Chi-square test results of failure mode proportions among groups (*)

	SB		SB		SB		CSEB		CSEB		CSEB		p
	Control		pH-cycling		Microbiological		Control		pH-cycling		Microbiological		
	24 h 12 mos		24 h 12 mos		24 h 12 mos		24 h 12 mos		24 h 12 mos		24 h 12 mos		
Adhesive/mixed	38 (79.2)	34 (72.3)	37 (78.7)	34 (73.9)	34 (70.8)	27 (57.4)	27 (65.9)	28 (71.8)	38 (86.4)	33 (76.7)	38 (77.5)	28 (58.3)	0.835
Cohesive in dentin	9 (18.7) a	9 (19.1) a	4 (8.5) a,c	3 (6.5) a,c	0 (0) b,c	1 (2.1) b,c	8 (19.5) a	9 (23.1) a	2 (4.5) b,c	2 (4.7) b,c	0 (0) b,c	1 (2.1)b,c	0.006
Cohesive in resin	1 (2.1)	4 (8.6)	0 (0)	0 (0)	2 (4.2)	0 (0)	6 (14.6)	2 (5.1)	1 (2.3)	0 (0)	1 (2.1)	0 (0)	0.177
Pre-testing failures	0 (0) b,c	0 (0) b,c	6 (12.8) a,d	8 (17.4) a,d	12 (25.0) d,e	19 (40.5) e	0 (0) b,c	0 (0) b,c	3 (6.8) a,c	8 (18.6) a,d	14 (20.4) d,e	19 (39.6) e	0.003
Total	48 (100)	47 (100)	47 (100)	46 (100)	48 (100)	47 (100)	41 (100)	39 (100)	44 (100)	43 (100)	49 (100)	48 (100)	

(*) Different letters indicate significant differences among groups (p < 0.05)

## Discussion

Based on the outcomes of the current study it can be stated that chemical and microbiological methods for caries-affected dentin induction resulted in lower μTBS values than that obtained to sound dentin. The rate of resin-dentin bond degradation after long-term of water aging was comparable between the methods, but more pronounced relative to sound substrate. These findings support the use of both methods of caries induction to simulate caries-affected dentin for testing the longevity of adhesive interfaces.

Previous investigations also demonstrated that the natural [17–19] or artificially-created CAD [5, 8, 20, 21] jeopardizes the bonding performance to this substrate. Greater number of porosities, lower mineral content [22] and reduced buffer capacity [23] verified in CAD results in a deeper zone of demineralized dentin that cannot completely be impregnated by resin monomers [2]. As consequence, there is a predominance of naked collagen fibrils denuded, making CAD interfaces more prone to deterioration over time [3].

Nonetheless, it is relevant to highlight that adhesive effectiveness verified in our study may somehow differ from the adhesive behavior on natural CAD, which presents the dentin tubules obliterated with acid-resistant whitlockite minerals as a response of the odontoblastic cells [10]. These precipitates limit the monomer infiltration and resin tag formation [17]. Despite that, the use of *in vitro* methodologies to induce standardized CAD surfaces may be more appropriate to perform laboratorial tests. In bond strength experiments a flat substrate is required to achieve the best interfacial loading orientation, which may be difficult considering the variability in size and shape of natural lesions.

The caries lesion induced by microbiological method seems to be quite more similar to the natural lesions, based on molecular and structural evaluations [24]. This model simulates the caries process using bacterial strains and reproduces features as color, texture change, collagen degradation and both zones found in natural dentin caries [25]. However, in our study, the percentage of pre-testing failures was higher in microbiological groups after 12 months of water storage, regardless of adhesive system tested.

This may be attributed to higher softening of the substrate created by the microbiological method, even after infected dentin layer removal, in comparison with natural or artificially-created CAD by pH-cycling model [10]. Additionally, this result can be also related to higher fragility of specimens’ preparation when performing microtensile test. Further studies using other design test, such as microshear test, are needed to confirm this hypothesis.

Otherwise, the pH-cycling model is more indicated to directly simulate a substrate that resembles CAD layer. Although this approach cannot reproduce all factors involved in natural caries process, its main advantage is the easier technique to create caries-like lesions compared to microbiological method, alternating the periods of demineralization and remineralization [26].

Regarding adhesive systems, bond strengths produced to sound dentin with CSEB were stable after water aging. Overall, reduction in bond strength for self-etch system to sound dentin was around 11 %, whereas for SB it was 42 %. The percentage reduction in μTBS values found in this study was next to ranges reported by Sanabe et al. [27] after 1 year of water exposure, considering the same materials tested. The resin–CAD bond longevity after longer aging period, as in our study, has been poorly investigated. Ricci et al. [7] verified that μTBS values decreased by 43.9 % after 12 months of oral function when an etch-and-rinse (Prime & Bond NT) was bonded to CAD in primary dentition.

The researches [28–30] using permanent teeth also have evidenced a greater resistance to degradation of resin-dentin bond with self-etch systems than those produced by etch-and-rinse adhesive systems. The use of a solvent-free adhesive layer [29], the creation of less defective hybrid layers [31] and the chemical interaction of MDP (10-methacryloxydecyl dihydrogen phosphate) with hidroxiapatite [32] may explain the superior durability of bond produced by CSEB.

## Conclusions

Both chemical and microbiological methods may be indicated to simulate caries-affected dentin for evaluating stability of resin-dentin bonds evaluations in primary teeth, irrespective of adhesive system.

### Availability of supporting data section

The data set supporting the results of this article are included within the paper and not as additional file.

## Abbreviations

SB: Adper Single Bond 2
CSEB: Clearfil SE Bond
μTBS: microtensile bond strength
ANOVA: Analysis of variance
CAD: Caries-affected dentin
